# Cholesterol Crystals and NLRP3 Mediated Inflammation in the Uterine Wall Decidua in Normal and Preeclamptic Pregnancies

**DOI:** 10.3389/fimmu.2020.564712

**Published:** 2020-10-08

**Authors:** Gabriela Brettas Silva, Lobke Marijn Gierman, Johanne Johnsen Rakner, Guro Sannerud Stødle, Siv Boon Mundal, Astrid Josefin Thaning, Bjørnar Sporsheim, Mattijs Elschot, Karin Collett, Line Bjørge, Marie Hjelmseth Aune, Liv Cecilie Vestrheim Thomsen, Ann-Charlotte Iversen

**Affiliations:** ^1^Centre of Molecular Inflammation Research, Department of Cancer Research and Molecular Medicine, Norwegian University of Science and Technology, Trondheim, Norway; ^2^Department of Gynecology and Obstetrics, St. Olavs Hospital, Trondheim, Norway; ^3^Department of Circulation and Medical Imaging, Norwegian University of Science and Technology, Trondheim, Norway; ^4^Department of Radiology and Nuclear Medicine, St. Olavs Hospital, Trondheim, Norway; ^5^Department of Pathology, Haukeland University Hospital, Bergen, Norway; ^6^Department of Gynecology and Obstetrics, Haukeland University Hospital, Bergen, Norway; ^7^Centre for Cancer Biomarkers CCBIO, Department of Clinical Science, University of Bergen, Bergen, Norway

**Keywords:** cholesterol crystals, decidua, fetal growth restriction, NLRP3 inflammasome, inflammation, IL-1β, placenta, preeclampsia

## Abstract

Preeclampsia is a hypertensive and inflammatory pregnancy disorder associated with cholesterol accumulation and inflammation at the maternal-fetal interface. Preeclampsia can be complicated with fetal growth restriction (FGR) and shares risk factors and pathophysiological mechanisms with cardiovascular disease. Cholesterol crystal mediated NLRP3 inflammasome activation is central to cardiovascular disease and the pathway has been implicated in placental inflammation in preeclampsia. Direct maternal-fetal interaction occurs both in the uterine wall decidua and at the placental surface and these aligned sites constitute the maternal-fetal interface. This study aimed to investigate cholesterol crystal accumulation and NLRP3 inflammasome expression by maternal and fetal cells in the uterine wall decidua of normal and preeclamptic pregnancies. Pregnant women with normal (*n* = 43) and preeclamptic pregnancies with (*n* = 28) and without (*n* = 19) FGR were included at delivery. Cholesterol crystals were imaged in decidual tissue by both second harmonic generation microscopy and polarization filter reflected light microscopy. Quantitative expression analysis of NLRP3, IL-1β and cell markers was performed by immunohistochemistry and automated image processing. Functional NLRP3 activation was assessed in cultured decidual explants. Cholesterol crystals were identified in decidual tissue, both in the tissue stroma and near uterine vessels. The cholesterol crystals in decidua varied between pregnancies in distribution and cluster size. Decidual expression of the inflammasome components NLRP3 and IL-1β was located to fetal trophoblasts and maternal leukocytes and was strongest in areas of proximity between these cell types. Pathway functionality was confirmed by cholesterol crystal activation of IL-1β in cultured decidual explants. Preeclampsia without FGR was associated with increased trophoblast dependent NLRP3 and IL-1β expression, particularly in the decidual areas of trophoblast and leukocyte proximity. Our findings suggest that decidual accumulation of cholesterol crystals may activate the NLRP3 inflammasome and contribute to decidual inflammation and that this pathway is strengthened in areas with close maternal-fetal interaction in preeclampsia without FGR.

## Introduction

Pregnancy is characterized by low-grade inflammation at the maternal-fetal interface and systemically in the mother, and this is aggravated to harmful levels in the pregnancy disorder preeclampsia (1). Preeclampsia is clinically characterized in the second half of pregnancy by new onset hypertension and proteinuria or maternal organ dysfunction and/or uteroplacental dysfunction (2, 3). The disease occurs in 4–5% of pregnancies and is a leading cause of maternal and fetal morbidity and mortality, often complicating with fetal growth restriction (FGR) (4). Preeclampsia is considered a warning sign for cardiovascular disease (CVD) later in life and common pathophysiological mechanisms are shared (5, 6). Cholesterol mediated inflammation has been suggested as a link between the disorders, a theory supported by preeclamptic pregnancies being characterized by a pro-atherogenic maternal lipid profile and cholesterol accumulation at the maternal-fetal interface (7, 8).

The uterine wall decidua and the placenta are the two aligned sites for direct maternal-fetal immunological interaction throughout pregnancy. In the decidua, specialized fetal cells, called extravillous trophoblasts, invade the tissue and establish a direct molecular dialogue with resident maternal cells such as decidual stromal cells and maternal immune cells (9). Leukocytes are key cells in modulating trophoblast behavior (10). Placental cytotrophoblasts fuse together to form a multinucleated cell layer, the syncytiotrophoblast, that covers the placenta and directly interacts with maternal blood. Preeclampsia and FGR are associated with reduced trophoblast invasion and impaired artery remodeling in the uterine wall, leading to placental oxidative stress and inflammation that increase as the fetus grows (11–13). Although significant progress has been made in understanding the central role of placental inflammation for development of preeclampsia and FGR (13–15), little is known about the involvement of inflammatory mechanisms in the decidua.

Intracellular crystallization of cholesterol is a complex process that occurs upon endocytosis of oxidized low-density lipoprotein (oxLDL) and this process has been extensively studied in the arterial wall in atherosclerosis (16, 17). The cholesterol crystals promote the development of atherosclerotic lesions by activation of the potent Nod-like receptor protein (NLRP)3 inflammasome (18). The resulting interleukin (IL)-1β production from this powerful activation may lead to extensive inflammation and tissue damage (15, 19). Trophoblasts express receptors that enable cholesterol transport and uptake of oxLDL has been shown to reduce trophoblast invasiveness (20–23), but cholesterol crystals have not been investigated at the maternal-fetal interface. Dysregulated lipid transport by reduced expression of ATP-binding cassette transporter (ABCA1) has been associated with cholesterol accumulation at the placental syncytiotrophoblast layer in preeclampsia (24) and in primary extravillous trophoblasts (25). Preeclampsia is associated with increased maternal systemic inflammatory markers including total cholesterol, oxLDL, IL-1β and soluble fms-like tyrosine kinase-1 (sFlt-1) (26–28). The NLRP3 inflammasome pathway in preeclampsia has been recently reviewed and mechanistically illustrated (29). We have previously shown that the NLRP3 inflammasome is active in the placenta and associated with preeclampsia, with a central involvement of trophoblasts (28). In the decidua, increased cholesterol accumulation in preeclampsia (7) and NLRP3 inflammasome expression in cultured cells (30) has been shown. This indicates a role for cholesterol crystal mediated NLRP3 inflammasome activation across the maternal-fetal interface, but the decidual involvement still needs to be determined.

We hypothesize that cholesterol accumulation in the decidua results in formation of cholesterol crystals, which induce decidual NLRP3 inflammasome activation and influence the important dialogue between trophoblasts and maternal immune cells. This study aimed to characterize cholesterol crystal accumulation and NLRP3 inflammasome expression by maternal and fetal cells in the uterine wall decidua of normal and preeclamptic pregnancies.

## Methods

### Study Participants and Decidual Biopsies

Women with normal and preeclamptic pregnancies with and without FGR were recruited at St. Olavs and Haukeland University Hospitals during 2002-2012. Preeclampsia was defined as persistent hypertension exceeding 140/90 mmHg plus proteinuria ≥0.3 g/24 h or ≥+1 by dipstick after 20 weeks of gestation. FGR was diagnosed by serial ultrasound measurements showing reduced intrauterine growth (*n* = 27), or, for neonates small for gestational age (*n* = 1), birth weight <5th percentile of Norwegian reference curves (31) combined with clinically and sonographically suspected FGR and/or postpartum defined placental pathology. Only singleton pregnancies undergoing cesarean section with no signs of labor were included. Decidua basalis tissue was collected by vacuum suction of the placental bed during cesarean section (7, 32). Tissue samples were either fixed in 10% neutral-buffered formalin and embedded in paraffin or snap frozen and stored at −80°C.

Placentas were collected from normal pregnancies after delivery by elective cesarean section for immediate isolation of decidual explants. The decidual tissue was dissected from the central region of the maternal side of the placenta. Samples were processed and cultured within 1.5 h after delivery.

### Decidual Explants

Decidual tissue was washed in sterile phosphate-buffered saline, cut into pieces (explants with wet weight range of 15–33 mg) and distributed in the culture plate evenly between the different culture conditions (24 ± 2 mg, mean ± standard deviation). There were no significant differences in explant weight between the culture conditions. Explants were cultured in Ham's F12/Dulbecco's modified Eagle's medium (DMEM) with 10% fetal bovine serum and 100 mg/mL penicillin-streptomycin (Sigma-Aldrich) and incubated for 24 h at 37°C, 8% O_2_ and 5% CO_2_ (33). Culture medium was then replaced by fresh culture medium with or without 500 pg/ml LPS priming (#tlrl-3pelps, InvivoGen, California, United States). After 2 h, the medium was replaced by fresh culture medium with or without stimuli; 200 or 2,000 μg/ml synthetic cholesterol crystals (#C3045, Sigma-Aldrich) or the positive control 3 mM ATP (#A7699, Sigma-Aldrich). Supernatants were collected after 24 h, centrifuged and stored at −80°C. Six technical replicates for each experimental condition were combined before analysis. Tissue viability was assessed by lactate dehydrogenase (LDH) cytotoxicity assay (#04744926001, Roche, Basel, Switzerland) (Supplementary Figure 1). IL-1β levels in supernatants were measured undiluted in duplicate using quantitative sandwich ELISA (#557953, BD Biosciences, New Jersey, United States).

### Cholesterol Crystal Imaging and Automated Quantification

**Figure 1:**
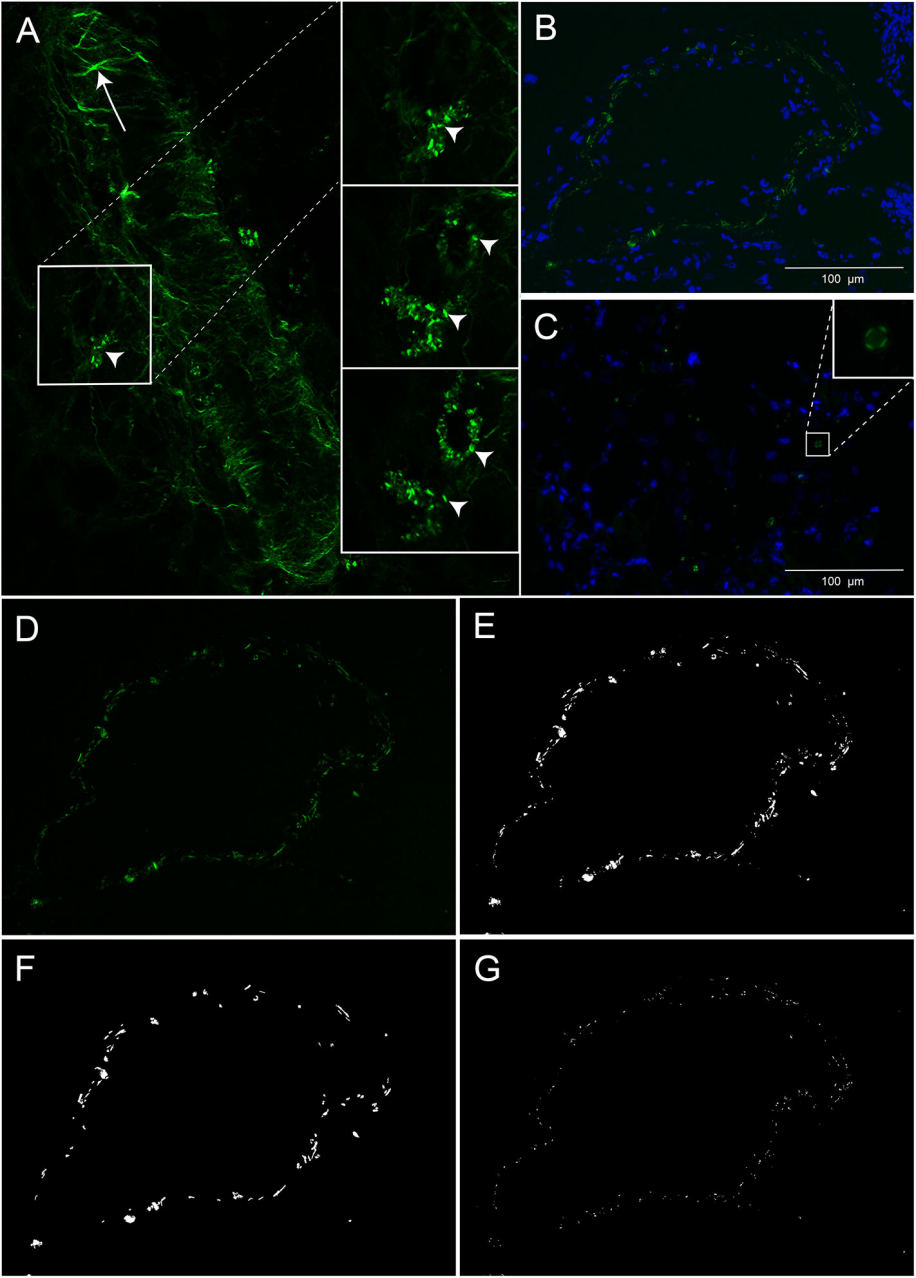
Cholesterol crystals in decidual tissue. **(A)** Decidual tissue imaged by second harmonic generation microscopy. The arrow heads indicate cholesterol crystals and the arrow points to collagen fibers in the vessel wall. **(B,C)** Decidual tissue stained by DAPI and imaged by polarized light microscopy. Nuclei (blue) and cholesterol crystals (green) are shown near a vessel wall **(B)** and within tissue stroma **(C)**. **(D–G)** Representative images obtained by polarized light microscopy for cholesterol crystal quantification. **(D)** Unprocessed image of cholesterol crystals (green). The same image processed by MATLAB showing **(E)** total cholesterol crystals and separated into **(F)** large and **(G)** small clusters of cholesterol crystals. Scale bar 100 μM.

To preserve cholesterol crystals, the decidual cryosections (5 μm) were analyzed untreated. DAPI mounting medium (#F6057, Sigma-Aldrich, Missouri, United States) was used to identify cellular nuclei. Cholesterol crystals in the decidual cryosections were first assessed by second harmonic generation microscopy (25X magnification Leica SP8 confocal microscope, Wetzlar; Germany). For further analysis of cholesterol crystals, three adjacent TIFF images (2,080 × 1,544 pixels) were obtained from three different regions in the decidual section using polarization filter reflected light microscopy (20X magnification, Inverted fluorescence microscope Olympus IX71, Tokyo, Japan) and defined microscope settings. The cholesterol crystals in the nine TIFF images per decidua were quantified by a customized MATLAB script (version 2018a, the MathWorks Inc., Massachusetts, United States) developed for automatic quantification of positive pixels. The total cholesterol crystal positive pixels in each decidua was determined by the sum of the positive pixels quantified in the nine pictures. The number of positive pixels in each of the nine images was used to assess the variation in cholesterol crystal tissue distribution within each decidua. To evaluate the size of the observed cholesterol crystals, aggregation of positive pixels was determined by defining small (<50 pixels) and large (≥50 pixels) cholesterol crystal clusters (representative image in Figures 1D–G). To verify that the crystals dissolved in alcohol, two serial decidual cryosections (5 μm) were obtained from four decidual tissue samples. One slide from each decidual sample was treated with PBS and the other slide was immersed in alcohol at room temperature for 60 s.

### Immunohistochemical Staining

Immunohistochemistry staining was performed in paraffin embedded decidua. Serial sections of 3 μm were pre-treated in PT link (#PT101, Dako, Glostrup, Denmark) using Target Retrieval Solution (#K8005 or #K8004, Dako) at 97°C for 20 min, and all sections were treated with peroxidase blocking solution (#K4007, Dako) or with dual peroxidase and alkaline phosphatase blocking solution (#K5361, Dako). Decidual tissue sections were incubated overnight at 4°C with antibodies against NLRP3 (1:100, #AG-20B-0014-C100, AdipoGen, California, United States) or IL-1β (1:200, #NB600-633, Novus, Colorado, United States); or for 45 min with cytokeratin 7 (CK7) (1:300, #M0851, Dako) or 40 min with CD45 (1:150, #M0701, Dako) at room temperature. All sections were incubated for 30 min with HRP-labeled polymer (#K4007, Dako) and for 10 min with DAB+ as chromogen (1:50, #K4007 or K5361, Dako). Decidual CK7 sections were double stained with smooth muscle actin (SMA) antibodies (1:300, #M0851, Dako) using EnVision G|2 Doublestain System Rabbit/Mouse (DAB+/Permanent Red) Kit system (K#5361, Dako). Overnight staining was performed manually and otherwise by Autostainer Plus (#S3800, Dako). Sections were counterstained with hematoxylin. Negative isotype controls were included (**Figures 3G,H**). In addition to the immunohistochemical staining, a routine staining with hematoxylin (#75290, Chemi-Teknik AS, Oslo, Norway), erythrosine 239 (#720-0179, VWR, Pennsylvania, United States) and saffron (#75100, Chemi-Teknik AS) (HES) was performed for each decidua using a Sakura Tissue-Tek © Prisma StainerTM (Sakura Finetek, Oslo, Norway).

### Automated Quantification of Protein Expression

Decidual tissue slides were scanned with the EVOS™ FL Auto Imaging System (Thermo Fisher Scientific, Massachusetts, United States), using 20X magnification and defined microscope settings. The large decidual scans varied in size depending on available tissue and consisted of 4 to 81 bright field TIFF images (2,048 × 1,536 pixels) per sample slide. A customized ImageJ (ImageJ_2_) (34, 35) script was used to perform background correction (Image calculator: Difference (img1 = |img1–img2|) and tile stitching [Grid/Collection stitching plugin (36)]. Smooth muscle tissue, placental tissue, blood vessels and tissue with poor morphology were excluded by manually defining regions of disinterest. NLRP3 and IL-1β expression was automatically quantified in the large tissue section scan for each decidua by a customized MATLAB script, and the protein expression quantified with examiners blinded to pregnancy outcomes. Cell specific staining was used to select decidual regions containing trophoblasts (CK7+) and leukocytes (CD45+). A mask of patches (1,325 × 1,325 μm) defining trophoblasts, maternal leukocytes, and maternal tissue without trophoblasts, was created for each decidual sample by using serial tissue section scans of cell-specific stained trophoblasts (CK7+) and leukocytes (CD45+). These masks were used to relate NLRP3 and IL-1β expression levels to trophoblasts and maternal leukocytes in the spatially aligned NLRP3 and IL-1β images. The *expression density* of CK7, CD45, NLRP3, and IL-1β in decidual tissue was calculated as the total number of positive stained pixels divided by the total amount of tissue pixels analyzed, to account for varying amounts of tissue between the samples. The *expression intensity* of NLRP3 and IL-1β were calculated as average staining intensity of all patches using a color deconvolution algorithm based on DAB specific RGB absorption (37).

### Statistical Methods

Statistical analyses were performed in SPSS (IBM SPSS Statistics 26, Illinois, United States) and GraphPad Prism (Prism8, California, United States). For clinical data, one-way ANOVA or Kruskal-Wallis with Tukey's or Dunn's multiple comparison *post hoc* test, respectively, were used for comparisons of continuous variables, and Fisher's exact test for categorical variables. Protein measurements in supernatants were analyzed by Kruskal-Wallis with Dunn's multiple comparison *post-hoc* test.

NLRP3 and IL-1β expression levels were compared between study groups using a linear mixed model with recruitment location, study group and the trophoblast and leukocyte densities implemented as fixed effects. Subject combinations and intercept were included as random effects. The cholesterol crystal analysis was performed by a linear mixed model with recruitment location and study group as fixed effect. Correlation between variables was performed by calculating Pearson's correlation coefficient. Alpha level was set to 0.05.

## Results

### Study Material

A total of 90 women with normal (*n* = 43) and preeclamptic pregnancies with (*n* = 28) and without FGR (*n* = 19) were included to the study (Table 1). The preeclamptic pregnancies with and without FGR, included more primiparas, had higher systolic and diastolic blood pressure, and their infants were delivered at earlier gestation with lower placental and birth weights, compared to normal pregnancies. The gestational age at delivery, as well as placenta and birth weights, were lower in preeclamptic pregnancies complicated with FGR compared to preeclamptic pregnancies without FGR (Table 1).

**Table 1:** Clinical characteristics of subjects included in third trimester decidual analyses (*n* = 90).

	**Normal pregnancies (*n* = 43)**	**Preeclampsia without FGR (*n* = 19)**	**Preeclampsia with FGR (*n* = 28)**
**Baseline characteristics**			
Maternal age, years	31.2 (± 5.4)^*^	29.3 (± 4,9)	29.5 (± 5.3)
Primiparas, n (%)	7 (16)	12 (63)^*^^||^	17 (61)^*^^||^
BMI^†^	24.8 (± 3.9)	25.7 (± 4.6)	25.4 (± 4.1)
**Characteristics at time of delivery**			
Systolic BP, mmHg^‡^	119 (± 10)	153 (± 20)^||^	148 (±18)^||^
Diastolic BP, mmHg^‡^	72 (± 8)	100 (± 12)^||^	96 (± 11)^||^
Severe preeclampsia, *n* (%)	n.a.	17 (89)	20 (69)
Early onset preeclampsia <34 weeks, *n* (%)	n.a.	15 (79)	21 (79)
Placental weight, g^§^	638 (102)	475 (128)^||^	290 (101)^||^^#^
Fetal birth weight, g	3,409 (332)	2,224 (583)^||^	1,311 (469)^||^^#^
Fetal sex, female, *n* (%)	23 (53.5)	8 (42.1)	16 (60.7)
Gestational age, weeks	38.6 (0.6)	33.6 (2.7)^||^	31.3 (3.1)^||^^#^

*FGR, fetal growth restriction; BMI, body mass index; BP, blood pressure; n.a., not applicable*.

*Continuous variables listed as means (± standard deviation) or median (interquartile range), assessed for differences between groups by one-way ANOVA with Tukey's post hoc test or Kruskal-Wallis with Dunn's post hoc test. Categorical variables listed as number (percent in column), assessed for differences between groups by Fisher's exact test*.

* *Information missing from one woman*.

† *Maternal BMI in first trimester. Information is missing from five women*.

‡ *Blood pressure from last healthcare visit before delivery. Information missing from one woman*.

§ *Information missing from 12 women*.

|| *P < 0.05 vs. normal pregnancies*.

# *P < 0.05 vs. preeclampsia without FGR*.

For the three pregnancies included in the decidual explant analysis, maternal age ranged between 31 and 38 years and gestational age between 38 and 39 weeks.

### Cholesterol Crystals

**Figure 2:**
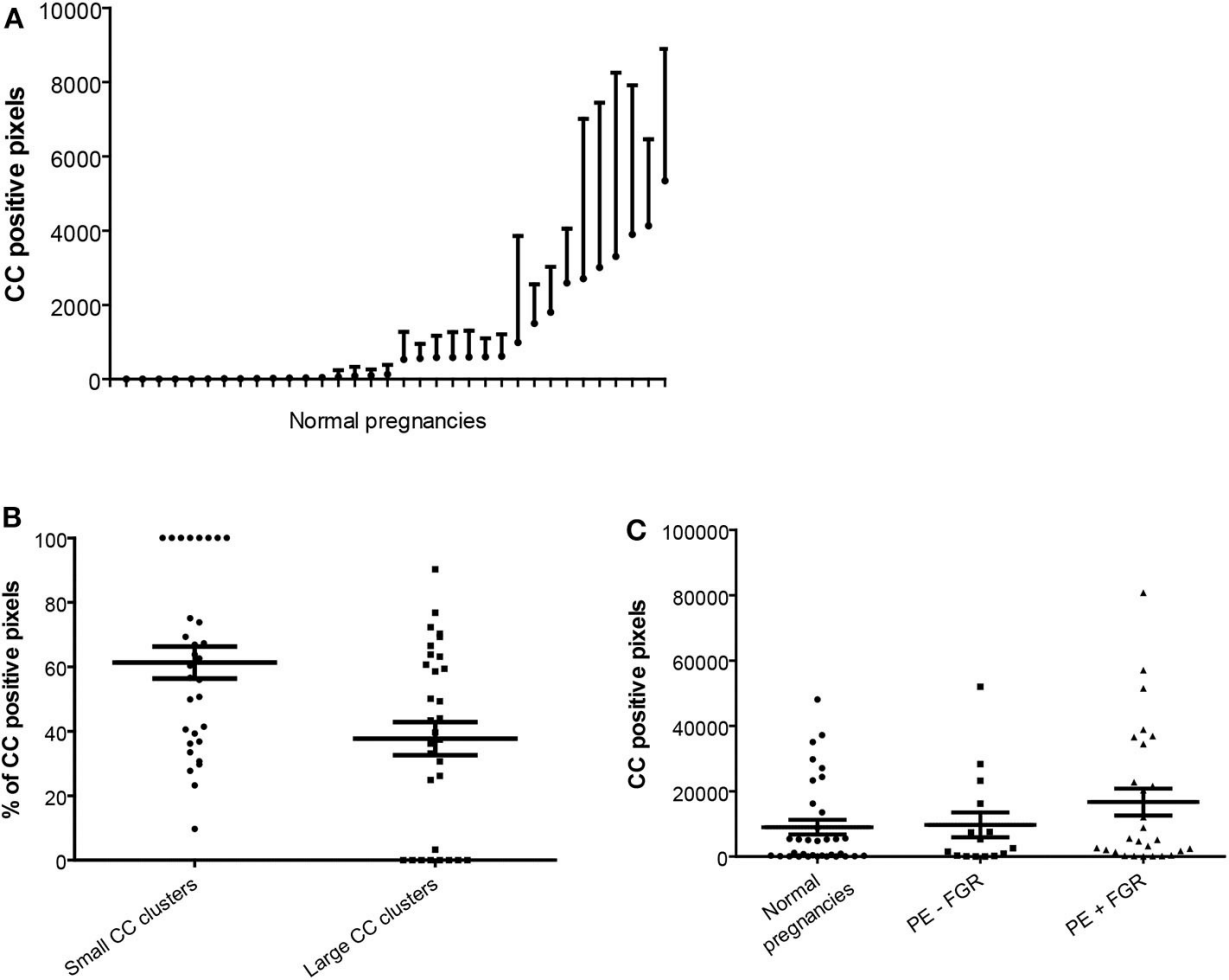
Quantification of cholesterol crystal in decidual tissue. Cholesterol crystal (CC) quantification was performed in nine pictures per decidual cryosection (5 μm). **(A)** Descriptive statistics of the cholesterol crystal positive pixels, shown as estimated means with standard error of mean, per normal pregnancy. **(B)** Percentages of the total cholesterol crystal positive pixels corresponding to small CC clusters (<50 pixels) and large CC clusters (≥50 pixels), in normal pregnancies. **(C)** Total cholesterol crystal positive pixels were quantified in decidual cryosections obtained from normal (*n* = 34) and preeclamptic pregnancies with (*n* = 27) and without fetal growth restriction (FGR) (*n* = 15).

Decidual cryosections from 76 women with normal (*n* = 34) and preeclamptic pregnancies with (*n* = 27) and without FGR (*n* = 15) were included. A marked presence of cholesterol crystals was observed dispersed in decidual tissue by both second harmonic generation (Figure 1A) and polarized light microscopy (Figures 1B–D). The crystals appeared to be localized both intra and extracellularly and cells containing cholesterol crystals were observed close to uterine vessels (Figure 1B) and within the tissue stroma (Figure 1C). The cholesterol crystals were not uniformly dispersed within the tissue stroma but were instead aggregated in distinct areas of the tissue and the distribution of such cholesterol crystal areas varied markedly between different pregnancies (Figure 2A). The cholesterol crystals appeared in the decidua as clusters of different size, and the ratio between large and small clusters in normal pregnancies was about 2:3 (Figure 2B). The cholesterol crystals detected in the decidua dissolved after alcohol treatment for 60 s, as expected (Supplementary Figure 2).

No significant differences in the amount, distribution and cluster size of decidual cholesterol crystals were detected between normal pregnancies and preeclamptic pregnancies with or without FGR (Figure 2C and data not shown).

### Decidual Tissue Composition

**Figure 3:**
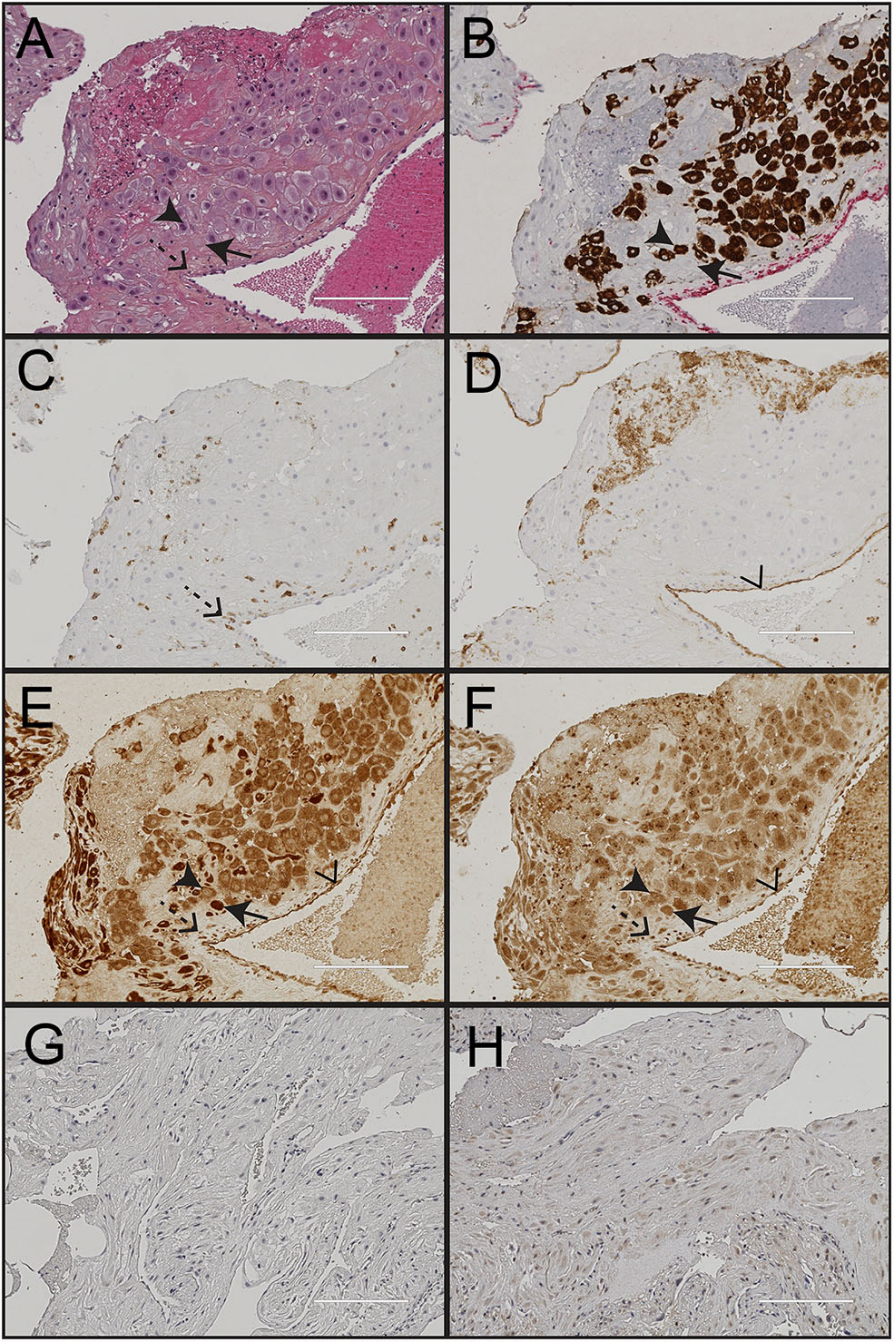
Immunohistochemical staining of decidua from a normal pregnancy. Representative images of decidual tissue from a normal pregnancy at gestational age 40 weeks. **(A)** HES; **(B)** the trophoblast marker cytokeratin 7 (CK7); **(C)** the endothelium marker CD31; **(D)** the leukocyte marker CD45; **(E)** nod-like receptor protein (NLRP)3; and **(F)** interleukin (IL)-1β. Negative isotype control shown for **(G)** NLRP3; and **(H)** IL-1β. Black arrowheads indicate trophoblasts, black arrows indicate maternal decidual stroma cells, dashed arrows indicate maternal leukocytes and transparent arrowhead indicate endothelial cells. Scale bar 200 μM.

Morphological assessment of cells and structures in the uterine wall decidua showed presence of fetal trophoblasts (CK7+), maternal leukocytes (CD45+), decidual stroma cells and uterine blood vessels (CD31+ endothelium) (Figures 3A–D). Trophoblasts were observed isolated or clustered in the tissue and were either apart from or in close contact with maternal leukocytes and decidual stroma cells. Both mononucleated trophoblasts and multinucleated trophoblast giant cells were identified and included in the analysis.

### Decidual NLRP3 Inflammasome Expression and Function

**Figure 4:**
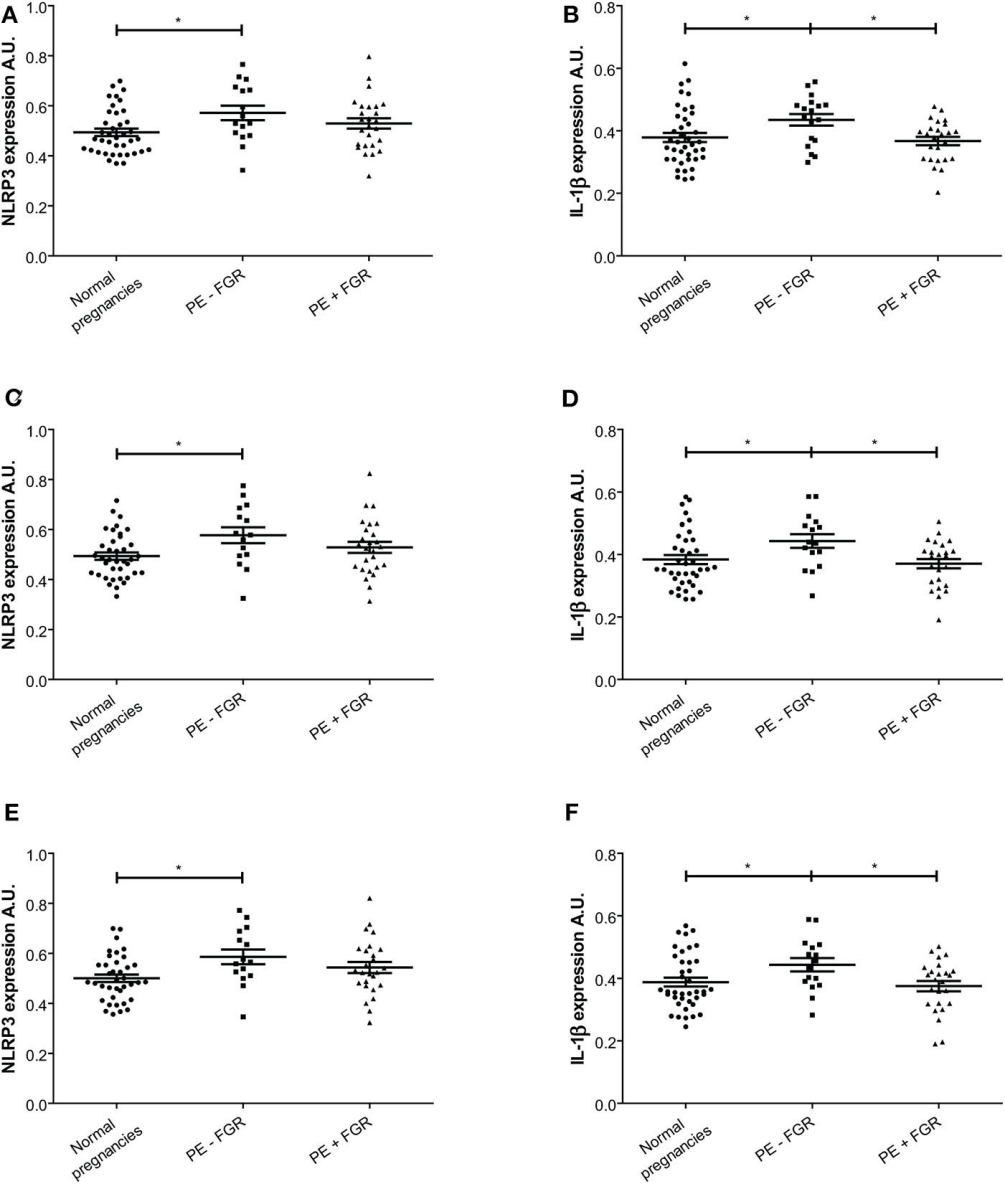
Expression intensity levels of nod-like receptor protein (NLRP)3 and interleukin (IL)-1β in decidual tissue. **(A,C,E)** NLRP3 expression intensity in decidual tissue from normal (*n* = 39) and preeclamptic pregnancies with (*n* = 26) and without fetal growth restriction (FGR) (*n* = 16); and **(B,D,F)** IL-1β expression intensity in normal (*n* = 40) and preeclamptic pregnancies with (*n* = 25) and without FGR (*n* = 18). Expression intensity is shown for **(A,B)** decidual tissue; **(C,D)** decidual areas containing trophoblasts; and **(E,F)** decidual areas containing trophoblast and leukocytes. Data were analyzed by a linear mixed model and expression levels are shown as estimated means with standard error of mean. *P < 0.05. A.U. indicates arbitrary units.

Paraffin embedded decidual tissue sections from 85 women with normal (*n* = 41) and preeclamptic pregnancies with (*n* = 26) and without FGR (*n* = 18) were included. From these, two pregnancies were excluded from the IL-1β and four from the NLRP3 expression analysis due to methodological errors in immunostaining or image processing. NLRP3 was strongly expressed in the cytoplasm of cells in the decidual tissue, including trophoblasts, maternal leukocytes, decidual stromal cells and endothelial cells (Figure 3E). The cell specific expression pattern of IL-1β in decidua was comparable to NLRP3 (Figure 3F). The cellular distribution and overall expression of NLRP3 and IL-1β in decidua of preeclamptic pregnancies appeared comparable to normal pregnancies (Supplementary Figure 3). The expression intensity (Figure 4, Supplementary Table 1) and density (Supplementary Table 2) of decidual NLRP3 and IL-1β were quantified. Preeclamptic pregnancies with normal fetal growth showed higher decidual expression intensity (Figures 4A,B) and density (Supplementary Table 2) of both NLRP3 and IL-1β compared to normal pregnancies (NLRP3 intensity *P* = 0.028 and density *P* = 0.006; IL-1β intensity *P* = 0.044, and density *P* = 0.010) and preeclamptic pregnancies complicated with FGR (NLRP3 intensity *P* = 0.372 and density *P* = 0.057; IL-1β intensity *P* = 0.024 and density *P* = 0.065, respectively). The increased decidual NLRP3 and IL-1β expression associated with preeclampsia with normal fetal growth could not be explained by differences in decidual leukocyte and trophoblast density (Supplementary Table 2). Preeclamptic pregnancies with FGR were associated with higher density of leukocytes, but not trophoblasts, compared to both preeclampsia with normal fetal growth and normal pregnancies (Supplementary Table 2).

A significant positive correlation between the decidual expression intensity of NLRP3 and IL-1β was observed in normal pregnancies (*R* = 0.516, *P* = 0.01) and pregnancies complicated with preeclampsia without FGR (*R* = 0.499, *P* = 0.05), but not in preeclamptic pregnancies with FGR (*R* = 0.323, *P* = 0.116). We have previously reported placental NLRP3 and IL-1β expression in 21 of the pregnancies included in this study (28). In this subgroup, we found no correlation between the decidual and placental expression intensity, neither in preeclampsia with (*n* = 5, NLRP3 *R* = −0.329, *P* = 0.588; IL-1β *R* = −0.579, *P* = 0.306) or without FGR (*n* = 10, NLRP3 *R* = 0.096, *P* = 0.791; IL-1β *R* = −0.251, *P* = 0.484), nor in normal pregnancies (*n* = 6, NLRP3 *R* = −0.718, *P* = 0.108; IL-1β *R* = −0.059, *P* = 0.912).

**Figure 5:**
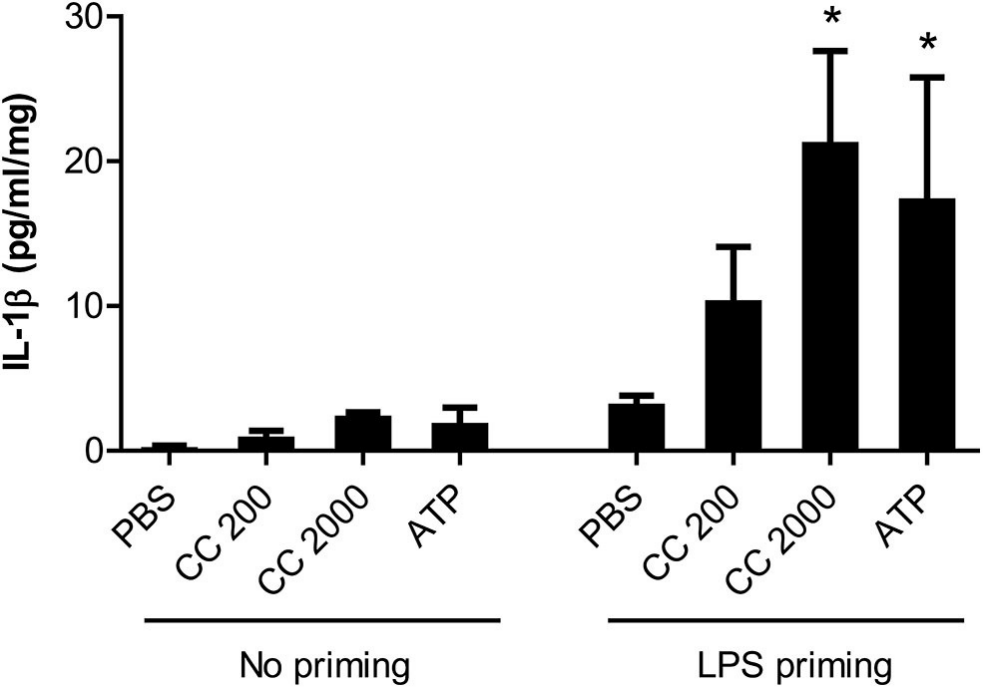
Interleukin (IL)-1β response following cholesterol crystal stimulation of decidual explants. Decidual explants from normal pregnancies (*n* = 3) were primed for 2 h with or without lipopolysaccharide (LPS), before adding cholesterol crystals (CC, 200 or 2,000 μg/ml) or adenosine triphosphate (ATP) for 24 h. Six technical replicates were included for each experimental condition. Release of IL-1β to the supernatant was measured by ELISA and presented as mean with standard error of mean, relative to explant weight. Data were analyzed using Kruskal-Wallis test with Dunn's multiple comparison *post-hoc* test. *P < 0.05. PBS, phosphate-buffered saline.

Synthetic cholesterol crystals induced NLRP3 inflammasome activation in LPS primed decidual tissue explants (*n* = 3) from normal pregnancies by significantly increasing the release of IL-1β (Figure 5). LDH cytotoxicity assay confirmed that the stimuli had no toxic effect on tissue viability (Supplementary Figure 1).

### Cellular NLRP3 Inflammasome Expression in Decidua

A significant positive correlation was found between the density of trophoblasts and the expression intensity of both NLRP3 (*R* = 0.244, *P* = 0.01) and IL-1β (*R* = 0.289, *P* = 0.01) in decidua. In decidual areas containing trophoblasts, both the NLRP3 and IL-1β expression intensity was significantly higher in preeclamptic pregnancies with normal fetal growth compared to normal pregnancies (NLRP3 *P* = 0.019; IL-1β *P* = 0.023) (Figures 4C,D). Further, the IL-1β expression intensity in trophoblast-containing decidual areas was significantly higher in preeclampsia without FGR compared to preeclampsia with FGR (*P* = 0.021) (Figure 4D). Excluding trophoblasts from analysis abolished the significant differences between the study groups (Supplementary Table 1).

The maternal leukocyte density in the decidua correlated weakly with the decidual expression intensity of NLRP3 (*R* = 0.044, *P* = 0.01) and IL-1β (*R* = 0.040, *P* = 0.01). Decidual tissue containing leukocytes showed higher expression intensity of NLRP3, but not IL-1β, in preeclamptic pregnancies with normal fetal growth compared to normal pregnancies (NLRP3 *P* = 0.025 and IL-1β *P* = 0.052) (Supplementary Table 1).

**Figure 6:**
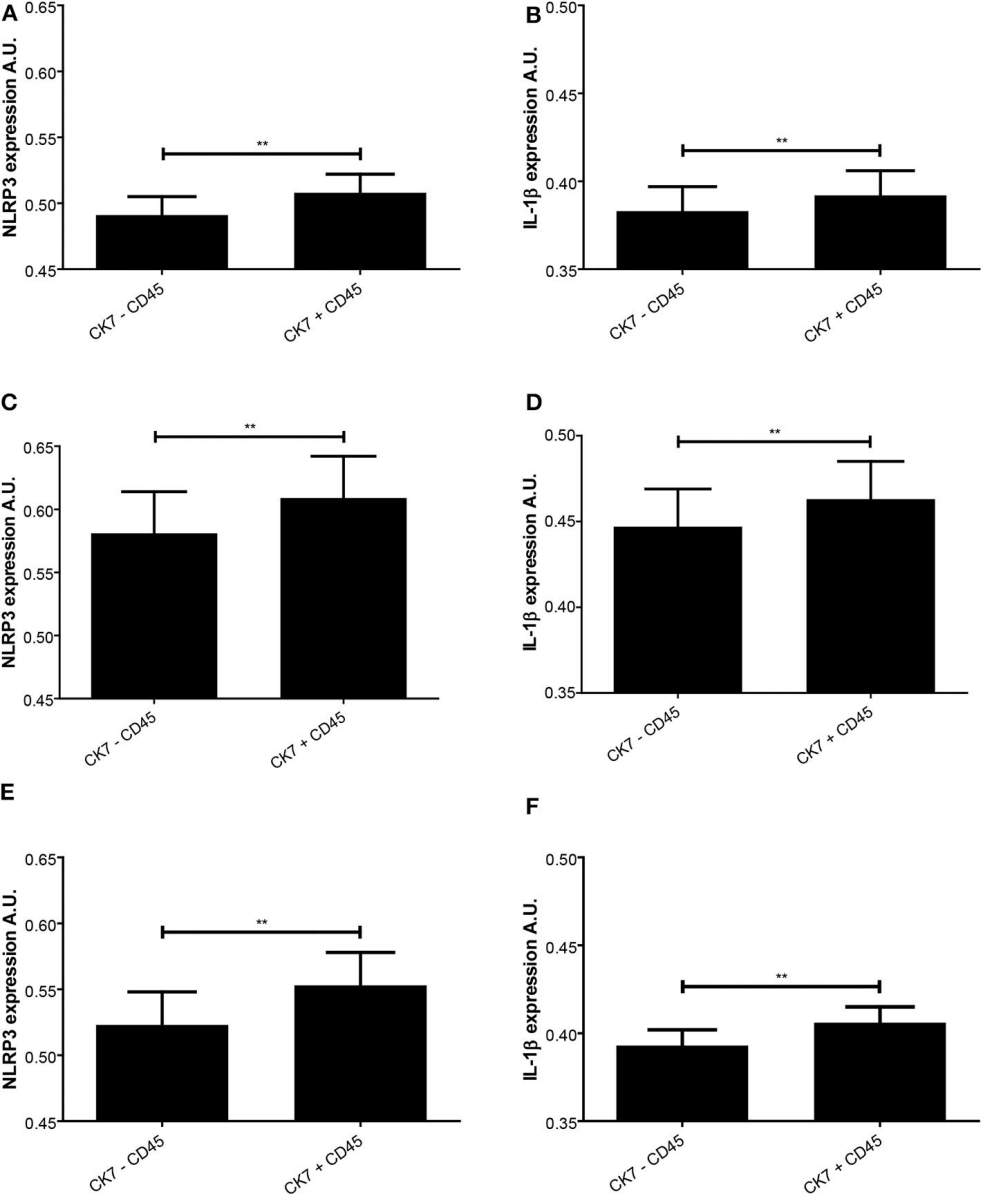
Expression intensity levels of nod-like receptor protein (NLRP)3 and interleukin (IL)-1β in decidual tissue related to presence of trophoblast and leukocytes. NLRP3 **(A,C,E)** and IL-1β **(B,D,F)** expression was measured in normal pregnancies **(A,B)** and preeclamptic pregnancies without **(C,D)** or with **(E,F)** fetal growth restriction (FGR). The expression levels were assessed in trophoblast containing areas with (CK7 + CD45) or without (CK7 – CD45) presence of leukocytes. **P < 0.01. A.U. indicates arbitrary units.

A closer look at decidual tissue with trophoblast and maternal leukocytes in proximity showed that the expression intensity of NLRP3 and IL-1β was significantly increased in preeclampsia without FGR compared to normal pregnancies (NLRP3 *P* = 0.017 and IL-1β *P* = 0.031) (Figures 4E,F). A comparison between trophoblast containing areas was made to assess the influence of leukocyte presence (Figure 6). Significantly higher expression intensity of both NLRP3 and IL-1β expression was observed in areas containing trophoblast and maternal leukocytes in proximity, compared to areas with trophoblasts and no leukocytes, and this dependence on maternal-fetal cell proximity was apparent in all study groups (Figure 6).

## Discussion

This study is the first to reveal the presence of cholesterol crystals at the maternal-fetal interface in the uterine wall decidua, and the crystals were shown to be markedly present in both normal and preeclamptic pregnancies. The cholesterol crystal responsive NLRP3 inflammasome and IL-1β were expressed by both fetal trophoblasts and maternal leukocytes in the decidua. Pathway functionality was confirmed by cholesterol crystal mediated activation of IL-1β production in decidual explants. The expression intensity levels of NLRP3 and IL-1β correlated within the decidua but not between the two sites of the maternal-fetal interface; decidua and placenta. Preeclampsia with normal fetal growth was associated with increased expression of NLRP3 and IL-1β, particularly in decidual areas of close maternal-fetal interaction.

Normal pregnancy is characterized by elevated maternal serum cholesterol and uric acid levels (7, 28, 38), and the correlation with increased serum levels of C-reactive protein (CRP) and sFlt-1 (28) indicates a potential contribution to the elevated maternal inflammatory state of normal pregnancies. The increased serum cholesterol may contribute to accumulation of cholesterol at the maternal-fetal interface (7), eventually leading to formation of cholesterol crystals in decidual tissue, as shown in the present study. Both trophoblasts and leukocytes are equipped with receptors that enable cholesterol uptake, such as the scavenger receptor CD36 (16, 21, 22, 39). Another aspect of how cholesterol crystals may contribute to decidual inflammation is linked to atherosis formation in uterine arteries, a vascular malformation resembling early stage atherosclerosis (40). CD36 is involved in macrophage foam cell formation and atherosclerosis progression by mediating endocytosis and conversion of oxLDL into cholesterol crystals, thus promoting complement and NLRP3 inflammasome activation (16, 41–43). Oxidative stress in the decidua may induce accumulation of oxLDL and cholesterol crystal formation. A similar role for cholesterol crystals in decidual macrophage foam cell accumulation and atherosis formation is supported by our observation of cholesterol crystals around the wall of uterine vessels, but further investigation is needed. We found that cells containing cholesterol crystals appeared aggregated within decidual tissue, rather than randomly scattered, suggesting that cholesterol crystal mediated inflammation may be localized to specific regions in the decidua. Previous studies have suggested that the size of cholesterol crystals and their clusters may be an important factor in the inflammatory potential (44, 45), but this hypothesis was not substantiated for decidual inflammation in the current study. The amount and size of cholesterol crystals in the decidua did not differ between normal and preeclamptic pregnancies. Further assessment of cell specific involvement in the decidual uptake and formation of cholesterol crystals, as well as involvement of relevant priming signals, such as complement factors (46), is warranted to fully understand the inflammatory potential of cholesterol crystals at the maternal-fetal interface. It must also be determined how different pathological processes in decidua interact since oxidative stress may promote the accumulation of oxLDL and cholesterol crystal formation (16, 17). The use of established advanced microscopy methodology for cholesterol imaging learned from atherosclerosis and hepatocytes lipid droplets (47–49), combined with removal of the crystals by alcohol treatment (50), strongly support that the imaged crystals in decidua are crystalline cholesterol. Still, further verification of the chemical identity of the crystals may be performed by other advanced microscopy techniques, such as coherent anti-Stokes Raman scattering (CARS) imaging.

This is the first demonstration of NLRP3 and IL-1β protein expression in maternal and fetal cells in the uterine wall decidua. A report of gene expression of NLRP3 inflammasome components in isolated first trimester decidual stromal cells partly supports our findings (30). Combined with our previous discovery of NLRP3 and IL-1β expression in placental trophoblasts of early and late pregnancies (28), this study clearly supports a role for NLRP3 inflammasome activation at both sites of the maternal-fetal interface throughout pregnancy. In addition, the positive correlation between decidual expression of NLRP3 and the responsive cytokine IL-1β substantiates the functionality of the NLRP3 inflammasome in decidua. Extravillous trophoblasts were here shown to be central for the decidual NLRP3 inflammasome response. This means that the characterization of placental trophoblasts and trophoblast cell lines as immunocompetent cells by their functional pattern recognition receptors (PRR) by us (28, 51, 52) and others (9, 53), has now been extended to extravillous trophoblasts in the decidua. This indicates importance for immunomodulating trophoblast activity at the maternal side of the maternal-fetal interface in the final stages of pregnancy, with dependence on the proximity and possible direct interaction between fetal and maternal cells. Supporting such maternal-fetal communication is that maternal leukocytes and trophoblasts in the decidua express complementary ligands and receptors (9, 10) and that leukocytes are key cells in modulating trophoblast behavior (54). In addition to leukocytes and trophoblasts, decidual stromal cells markedly expressed NLRP3 and IL-1β and are considered potential responders to cholesterol crystals, but further studies focused on this cell type are needed to address their inflammatory role.

Preeclampsia without FGR was associated with increased decidual expression of NLRP3 and IL-1β, suggesting that the NLRP3 inflammasome aggravates the inflammatory response and substantiates the reported shift to a pro-inflammatory profile and cell type distribution at the maternal-fetal interface in preeclampsia (9, 14, 29). The increased decidual inflammasome expression was trophoblast dependent and strongest in areas where trophoblast and leukocytes are in proximity, suggesting that NLRP3 mediated inflammation may disturb maternal-fetal communication. The novel identification of decidual cholesterol crystals combined with increased NLRP3 inflammasome expression presents a novel link between the pathophysiology of CVD and preeclampsia. NLRP3 inflammasome response may lead to extensive tissue damage and cell death and is associated with formation and progression of atherosclerotic lesions (18, 19). Correspondingly, increased formation of decidual atherosis and inflammation at the maternal-fetal interface are pathophysiological features of preeclampsia (40, 55). The NLRP3 inflammasome expression pattern in decidua further points to interesting pathophysiological differences between preeclampsia subgroups. We have previously demonstrated a placental role for the NLRP3 inflammasome in preeclampsia combined with FGR (28), while the decidual contribution presented here was apparent in preeclampsia without FGR. This points to divergent NLRP3 inflammasome activation in preeclampsia subgroups. Supporting such divergent regulation is the lack of correlation between decidual and placental expression levels of NLRP3 and IL-1β, indicating that activators in the maternal serum affect placental and decidual tissue differentially and that the local inflammatory responses in the decidua and placenta are not directly coordinated. We hypothesize that the placental tissue may be more influenced by placental dysfunction and fetal complications. FGR has been shown associated to a fetal pro-atherogenic lipid profile and placental cholesterol accumulation due to abnormal cholesterol transport (24, 56). This may lead to cholesterol accumulation and crystallization in the placenta and activation of the NLRP3 inflammasome in a process that may not influence decidual tissue. In the present study, increased decidual NLRP3 inflammasome response was observed in preeclampsia without FGR, and this could indicate that the decidual tissue may respond more to the increased maternal danger signals, including pro-atherogenic lipid profile, circulating levels of inflammatory mediators, such as HMGB1 and uric acid, and predisposition to inflammation.

Therapeutic and preventive approaches in preeclampsia are limited. A large prospective study showed limited preventive effect of administration of low-dose anti-inflammatory and antiplatelet agent aspirin on preeclampsia development (57). Pravastatin, used for treatment of dyslipidemia and prevention of CVD, is a suggested candidate for treatment and prevention of preeclampsia (58). In addition to reducing hypercholesterolemia, statins may ameliorate major pathological responses involved in preeclampsia, including inhibition of sFlt-1 release and reduction of inflammation and oxidative stress (59). Importantly, statins may inhibit formation and improve solubility of cholesterol crystals in atherosclerotic plaques (60), adding to the beneficial effects of these cholesterol-lowering drugs. Further investigation is needed to demonstrate whether a positive effect of pravastatin in preeclampsia may involve removal of cholesterol crystals at the maternal-fetal interface. Growing evidence supports the clinical benefits and anti-inflammatory effects of drugs targeting the NLRP3 inflammasome and IL-1β pathway in several diseases (61), but whether they are pregnancy-safe and effective in preeclampsia needs to be determined.

This study identified cholesterol crystal mediated NLRP3 inflammasome response as an inflammatory mechanism associated with maternal-fetal interaction in the uterine wall decidua in pregnancy. Cholesterol crystals were detected in considerable amounts in decidua and the expression of the NLRP3 inflammatory pathway showed importance for close interaction between fetal trophoblasts and maternal leukocytes. The increased NLRP3 inflammasome expression in preeclampsia with normal fetal growth suggests that an enhanced pro-inflammatory profile in the decidua negatively affects maternal-fetal communication and plays a role at late stages of preeclampsia pathology, possibly by intercommunication with the maternal systemic inflammatory response. The identification of decidual cholesterol crystals and increased decidual NLRP3 inflammasome expression in preeclampsia with normal fetal growth further substantiates the pathophysiological link between preeclampsia and CVD. This study showed that combined investigation of cell specific pathological mechanisms at the two sites of maternal-fetal interface may provide more comprehensive knowledge of the regulation and importance of maternal-fetal communication.

## Data Availability Statement

The data that support the findings of this study are available from the corresponding author upon reasonable request.

## Ethics Statement

The Norwegian Regional Committee for Medical and Health Research Ethics (REC) approved the study (REC 2012/1040 and 2009/03) and written informed consent was obtained from participants. Procedures were in accordance with institutional guidelines. The patients/participants provided their written informed consent to participate in this study.

## Author Contributions

GBS, LG, and A-CI designed the study and all authors contributed with valuable inputs. GBS, LT, and LB collected the clinical material and information. GBS, LG, JR, SM, AT, ME, and A-CI developed the methods for image processing and automated quantification. GBS and GSS performed the decidual explant experiments. GBS, LG, and A-CI interpreted the data and wrote the manuscript. All authors contributed to the article and approved the submitted version.

## Conflict of Interest

The authors declare that the research was conducted in the absence of any commercial or financial relationships that could be construed as a potential conflict of interest.

## References

[1] Redman CWG Sacks GP Sargent IL. Preeclampsia: an excessive maternal inflammatory response to pregnancy. Am J Obstetr Gynecol. (1999) 180:499–506. 10.1016/S0002-9378(99)70239-5 9988826

[2] Staff AC Andersgaard AB Henriksen T Langesæter E Magnussen E Michelsen TM. Chapter 28 Hypertensive disorders of pregnancy and eclampsia. Eur J Obstetr Gynecol Reprod Biol. (2016) 201:171–8. 10.1016/j.ejogrb.2016.04.001 27160502

[3] Brown MA Magee LA Kenny LC Karumanchi SA McCarthy FP Saito S. The hypertensive disorders of pregnancy: ISSHP classification, diagnosis & management recommendations for international practice. Preg Hypertens. (2018) 13:291–310. 10.1016/j.preghy.2018.05.004 29803330

[4] Abalos E Cuesta C Grosso AL Chou D Say L. Global and regional estimates of preeclampsia and eclampsia: a systematic review. Eur J Obstetr Gynecol Reprod Biol. (2013) 170:1–7. 10.1016/j.ejogrb.2013.05.005 23746796

[5] Bellamy L Casas J-P Hingorani AD Williams DJ. Pre-eclampsia and risk of cardiovascular disease and cancer in later life: systematic review and meta-analysis. BMJ. (2007) 335:974. 10.1136/bmj.39335.385301.BE 17975258 PMC2072042

[6] Brown MC Best KE Pearce MS Waugh J Robson SC Bell R. Cardiovascular disease risk in women with pre-eclampsia: systematic review and meta-analysis. Eur J Epidemiol. (2013) 28:1–19. 10.1007/s10654-013-9762-6 23397514

[7] Staff AC Ranheim T Khoury J Henriksen T. Increased contents of phospholipids, cholesterol, and lipid peroxides in decidua basalis in women with preeclampsia. Am J Obstetr Gynecol. (1999) 180:587–92. 10.1016/S0002-9378(99)70259-0 10076133

[8] Adank MC Benschop L Peterbroers KR Smak Gregoor AM Kors AW Mulder MT. Is maternal lipid profile in early pregnancy associated with pregnancy complications and blood pressure in pregnancy and long term postpartum? Am J Obstetr Gynecol. (2019) 221:150.e151–13. 10.1016/j.ajog.2019.03.025 30940559

[9] Vento-Tormo R Efremova M Botting RA Turco MY Vento-Tormo M Meyer KB. Single-cell reconstruction of the early maternal–fetal interface in humans. Nature. (2018) 563:347–53. 10.1038/s41586-018-0698-6 30429548 PMC7612850

[10] Menkhorst EM Van Sinderen M Correia J Dimitriadis E. Trophoblast function is altered by decidual factors in gestational-dependant manner. Placenta. (2019) 80:8–11. 10.1016/j.placenta.2019.03.013 31103068

[11] Redman CWG Sargent IL. Review article: immunology of pre-eclampsia. Am J Reprod Immunol. (2010) 63:534–43. 10.1111/j.1600-0897.2010.00831.x 20331588

[12] Brosens I Pijnenborg R Vercruysse L Romero R. The “Great Obstetrical Syndromes” are associated with disorders of deep placentation. Am J Obstetr Gynecol. (2011) 204:193–201. 10.1016/j.ajog.2010.08.009 21094932 PMC3369813

[13] Chaiworapongsa T Chaemsaithong P Yeo L Romero R. Pre-eclampsia part 1: current understanding of its pathophysiology. Nat Rev Nephrol. (2014) 10:466–80. 10.1038/nrneph.2014.102 25003615 PMC5893150

[14] Harmon AC Cornelius DC Amaral LM Faulkner JL Cunningham MW Wallace K. The role of inflammation in the pathology of preeclampsia. Clin Sci. (2016) 130:409–19. 10.1042/CS20150702 26846579 PMC5484393

[15] Gomez-Lopez N Motomura K Miller D Garcia-Flores V Galaz J Romero R. Inflammasomes: their role in normal and complicated pregnancies. J Immunol. (2019) 203:2757–69. 10.4049/jimmunol.1900901 31740550 PMC6871659

[16] Sheedy FJ Grebe A Rayner KJ Kalantari P Ramkhelawon B Carpenter SB. CD36 coordinates NLRP3 inflammasome activation by facilitating intracellular nucleation of soluble ligands into particulate ligands in sterile inflammation. Nat Immunol. (2013) 14:812–20. 10.1038/ni.2639 23812099 PMC3720827

[17] Franklin BS Mangan MS Latz E. Crystal formation in inflammation. Ann Rev Immunol. (2016) 34:173–202. 10.1146/annurev-immunol-041015-055539 26772211

[18] Duewell P Kono H Rayner KJ Sirois CM Vladimer G Bauernfeind FG. NLRP3 inflammasomes are required for atherogenesis and activated by cholesterol crystals. Nature. (2010) 464:1357–61. 10.1038/nature08938 20428172 PMC2946640

[19] Grebe A Hoss F Latz E. NLRP3 inflammasome and the IL-1 pathway in atherosclerosis. Circ Res. (2018) 122:1722–40. 10.1161/CIRCRESAHA.118.311362 29880500

[20] Pavan L Tsatsaris V Hermouet A Therond P Evain-Brion D Fournier T. Oxidized low-density lipoproteins inhibit trophoblastic cell invasion. J Clin Endocrinol Metab. (2004) 89:1969–72. 10.1210/jc.2003-032042 15070971

[21] Plösch T van Straten EME Kuipers F. Cholesterol transport by the placenta: placental liver X receptor activity as a modulator of fetal cholesterol metabolism? Placenta. (2007) 28:604–10. 10.1016/j.placenta.2006.10.009 17141866

[22] Duttaroy AK. Transport of fatty acids across the human placenta: a review. Progr Lipid Res. (2009) 48:52–61. 10.1016/j.plipres.2008.11.001 19041341

[23] Woollett LA. Review: transport of maternal cholesterol to the fetal circulation. Placenta. (2011) 32:S218–21. 10.1016/j.placenta.2011.01.011 21300403 PMC4699659

[24] Baumann M Körner M Huang X Wenger F Surbek D Albrecht C. Placental ABCA1 and ABCG1 expression in gestational disease: pre-eclampsia affects ABCA1 levels in syncytiotrophoblasts. Placenta. (2013) 34:1079–86. 10.1016/j.placenta.2013.06.309 23880356

[25] Vondra S Kunihs V Eberhart T Eigner K Bauer R Haslinger P. Metabolism of cholesterol and progesterone is differentially regulated in primary trophoblastic subtypes and might be disturbed in recurrent miscarriages. J Lipid Res. (2019) 60:1922–34. 10.1194/jlr.P093427 31530576 PMC6824492

[26] Koçyigit Y Atamer Y Atamer A Tuzcu A Akkus Z. Changes in serum levels of leptin, cytokines and lipoprotein in pre-eclamptic and normotensive pregnant women. Gynecol Endocrinol. (2004) 19:267–73. 10.1080/09513590400018108 15726915

[27] Levine RJ Lam C Qian C Yu KF Maynard SE Sachs BP. Soluble endoglin and other circulating antiangiogenic factors in preeclampsia. New Engl J Med. (2006) 355:992–1005. 10.1056/NEJMoa055352 16957146

[28] Stødle GS Silva GB Tangerås LH Gierman LM Nervik I Dahlberg UE. Placental inflammation in pre-eclampsia by Nod-like receptor protein (NLRP)3 inflammasome activation in trophoblasts. Clin Exp Immunol. (2018) 193:84–94. 10.1111/cei.13130 29683202 PMC6038006

[29] Shirasuna K Karasawa T Takahashi M. Role of the NLRP3 inflammasome in preeclampsia. Front Endocrinol. (2020) 11. 10.3389/fendo.2020.00080 32161574 PMC7053284

[30] Pontillo A Girardelli M Agostinis C Masat E Bulla R Crovella S. Bacterial LPS differently modulates inflammasome gene expression and IL-1β secretion in trophoblast cells, decidual stromal cells, and decidual endothelial cells. Reprod Sci. (2013) 20:563–6. 10.1177/1933719112459240 23184659

[31] Johnsen SL Rasmussen S Wilsgaard TOM Sollien R Kiserud T. Longitudinal reference ranges for estimated fetal weight. Acta Obstetr Gynecol Scand. (2006) 85:286–97. 10.1080/00016340600569133 16553175

[32] Harsem NK Staff AC He L Roald B. The decidual suction method: a new way of collecting decidual tissue for functional and morphological studies. Acta Obstet Gynecol Scand. (2004) 83:724–30. 10.1111/j.0001-6349.2004.00395.x 15255844

[33] Miller RK Genbacev O Turner MA Aplin JD Caniggia I Huppertz B. Human placental explants in culture: approaches and assessments. Placenta. (2005) 26:439–48. 10.1016/j.placenta.2004.10.002 15950058

[34] Schindelin J Arganda-Carreras I Frise E Kaynig V Longair M Pietzsch T. Fiji: an open-source platform for biological-image analysis. Nat Methods. (2012) 9:676–82. 10.1038/nmeth.2019 22743772 PMC3855844

[35] Rueden CT Schindelin J Hiner MC DeZonia BE Walter AE Arena ET. ImageJ2: ImageJ for the next generation of scientific image data. BMC Bioinform. (2017) 18:529. 10.1186/s12859-017-1934-z 29187165 PMC5708080

[36] Preibisch S Saalfeld S Tomancak P. Globally optimal stitching of tiled 3D microscopic image acquisitions. Bioinformatics. (2009) 25:1463–5. 10.1093/bioinformatics/btp184 19346324 PMC2682522

[37] Ruifrok AC Johnston DA. Quantification of histochemical staining by color deconvolution. Anal Quant Cytol Histol. (2001) 23:291–9. 11531144

[38] Lippi G Albiero A Montagnana M Salvagno GL Scevarolli S Franchi M. Lipid and lipoprotein profile in physiological pregnancy. Clin Lab. (2007) 53:173–7. 17447654

[39] Horne H Holme AM Roland MCP Holm MB Haugen G Henriksen T. Maternal-fetal cholesterol transfer in human term pregnancies. Placenta. (2019) 87:23–9. 10.1016/j.placenta.2019.09.001 31541855

[40] Alnaes-Katjavivi P Lyall F Roald B Redman CWG Staff AC. Acute atherosis in vacuum suction biopsies of decidua basalis: an evidence based research definition. Placenta. (2016) 37:26–33. 10.1016/j.placenta.2015.10.020 26608629

[41] Park YM. CD36, a scavenger receptor implicated in atherosclerosis. Exp Mol Med. (2014) 46:e99. 10.1038/emm.2014.38 24903227 PMC4081553

[42] Niyonzima N Halvorsen B Sporsheim B Garred P Aukrust P Mollnes TE. Complement activation by cholesterol crystals triggers a subsequent cytokine response. Mol Immunol. (2017) 84:43–50. 10.1016/j.molimm.2016.09.019 27692470

[43] Gravastrand CS Steinkjer B Halvorsen B Landsem A Skjelland M Jacobsen EA. Cholesterol crystals induce coagulation activation through complement-dependent expression of monocytic tissue factor. J Immunol. (2019) 203:853. 10.4049/jimmunol.1900503 31270150 PMC6680065

[44] Abela GS Kalavakunta JK Janoudi A Leffler D Dhar G Salehi N. Frequency of cholesterol crystals in culprit coronary artery aspirate during acute myocardial infarction and their relation to inflammation and myocardial injury. Am J Cardiol. (2017) 120:1699–707. 10.1016/j.amjcard.2017.07.075 28867129

[45] Shu F Chen J Ma X Fan Y Yu L Zheng W. Cholesterol crystal-mediated inflammation is driven by plasma membrane destabilization. Front Immunol. (2018) 9:1163. 10.3389/fimmu.2018.01163 29896195 PMC5986904

[46] Samstad EO Niyonzima N Nymo S Aune MH Ryan L Bakke SS. Cholesterol crystals induce complement-dependent inflammasome activation and cytokine release. J Immunol. (2014) 192:2837–45. 10.4049/jimmunol.1302484 24554772 PMC3985066

[47] James J Tanke HJ Biomedical Light Microscopy. Amsterdam: Springer Science & Business Media (2012).

[48] Suhalim JL Chung CY Lilledahl MB Lim RS Levi M Tromberg BJ. Characterization of cholesterol crystals in atherosclerotic plaques using stimulated raman scattering and second-harmonic generation microscopy. Biophys J. (2012) 102:1988–95. 10.1016/j.bpj.2012.03.016 22768956 PMC3328706

[49] Ioannou GN Subramanian S Chait A Haigh WG Yeh MM Farrell GC. Cholesterol crystallization within hepatocyte lipid droplets and its role in murine NASH. J Lipid Res. (2017) 58:1067–79. 10.1194/jlr.M072454 28404639 PMC5456359

[50] Nasiri M Janoudi A Vanderberg A Frame M Flegler C Flegler S. Role of cholesterol crystals in atherosclerosis is unmasked by altering tissue preparation methods. Microsc Res Techn. (2015) 78:969–74. 10.1002/jemt.22560 26278962

[51] Tangerås LH Stødle GS Olsen GD Leknes A-H Gundersen AS Skei B. Functional Toll-like receptors in primary first-trimester trophoblasts. J Reprod Immunol. (2014) 106:89–99. 10.1016/j.jri.2014.04.004 24933117

[52] Gierman LM Stødle GS Tangerås LH Austdal M Olsen GD Follestad T. Toll-like receptor profiling of seven trophoblast cell lines warrants caution for translation to primary trophoblasts. Placenta. (2015) 36:1246–53. 10.1016/j.placenta.2015.09.004 26386649

[53] Guleria I Pollard JW. The trophoblast is a component of the innate immune system during pregnancy. Nat Med. (2000) 6:589–93. 10.1038/75074 10802718

[54] Ander SE Diamond MS Coyne CB. Immune responses at the maternal-fetal interface. Sci Immunol. (2019) 4:eaat6114. 10.1126/sciimmunol.aat6114 30635356 PMC6744611

[55] Khong TY Mooney EE Ariel I Balmus NCM Boyd TK Brundler M-A. Sampling and definitions of placental lesions: amsterdam placental workshop group consensus statement. Arch Pathol Lab Med. (2016) 140:698–713. 10.5858/arpa.2015-0225-CC 27223167

[56] Barker DJ. Adult consequences of fetal growth restriction. Clin Obstetr Gynecol. (2006) 49:270–83. 10.1097/00003081-200606000-00009 16721106

[57] Rolnik DL Wright D Poon LC O'Gorman N Syngelaki A de Paco Matallana C. Aspirin versus placebo in pregnancies at high risk for preterm preeclampsia. New Engl J Med. (2017) 377:613–22. 10.1056/NEJMoa1704559 28657417

[58] Maierean SM Mikhailidis DP Toth PP Grzesiak M Mazidi M Maciejewski M. The potential role of statins in preeclampsia and dyslipidemia during gestation: a narrative review. Exp Opin Investig Drugs. (2018) 27:427–35. 10.1080/13543784.2018.1465927 29672173

[59] Ramma W Ahmed A Therapeutic potential of statins and the induction of heme oxygenase-1 in preeclampsia. J Reprod Immunol. (2014) 101–2:153–60. 10.1016/j.jri.2013.12.120 PMC4003533 24503248

[60] Abela GS Vedre A Janoudi A Huang R Durga S Tamhane U. Effect of statins on cholesterol crystallization and atherosclerotic plaque stabilization. Am J Cardiol. (2011) 107:1710–7. 10.1016/j.amjcard.2011.02.336 21507364

[61] Shao B-Z Cao Q Liu C. Targeting NLRP3 inflammasome in the treatment of CNS diseases. Front Mol Neurosci. (2018) 11:320. 10.3389/fnmol.2018.00320 30233319 PMC6131647

