# Supplementary figures and images for: Cholesterol Crystals and NLRP3 Mediated Inflammation in the Uterine Wall Decidua in Normal and Preeclamptic Pregnancies
Source: Front Immunol. 2020 Oct 8;11:564712. doi: 10.3389/fimmu.2020.564712 (PMC7578244; doi:10.3389/fimmu.2020.564712)

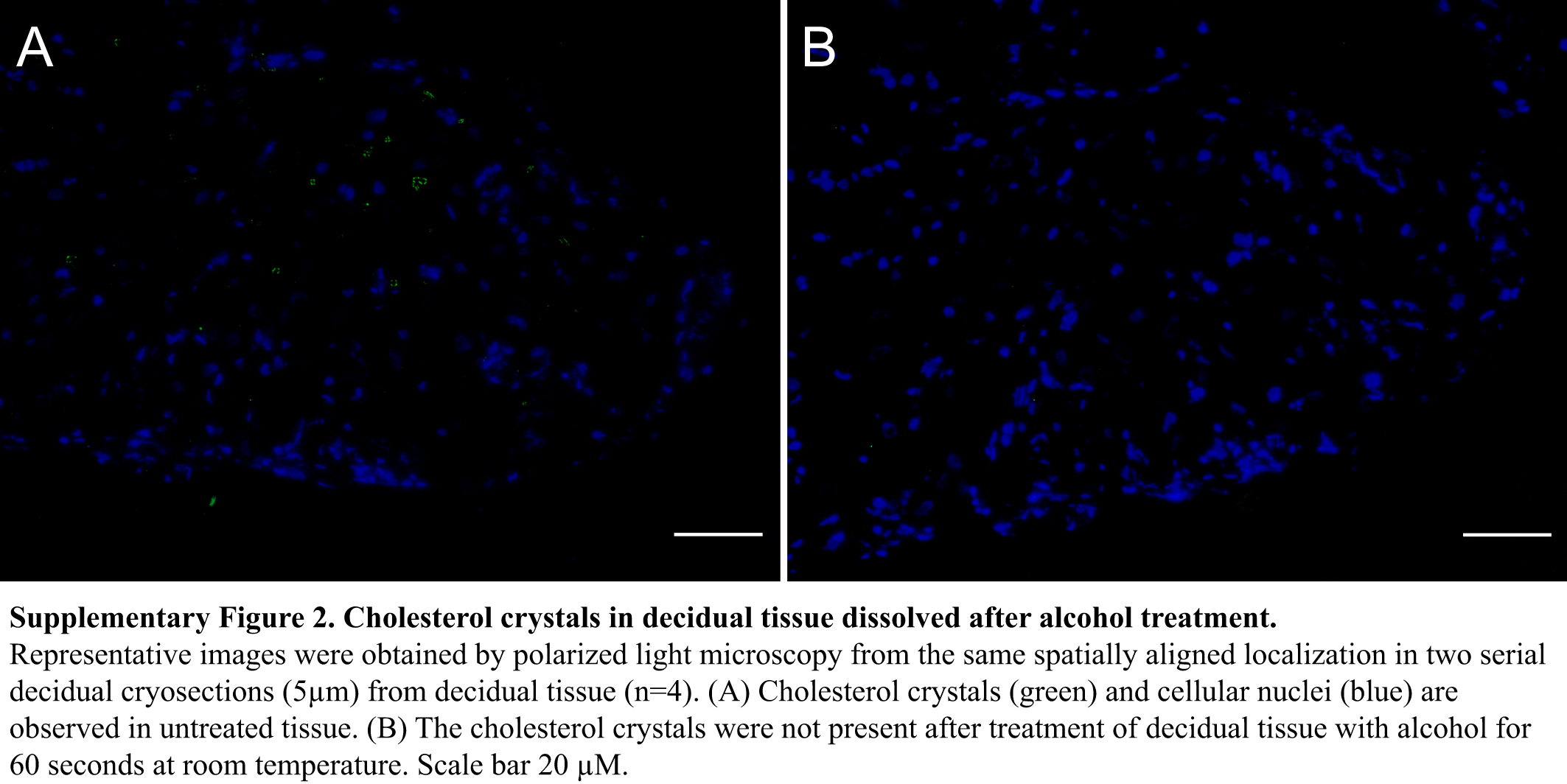

Supplement: Supplementary file 2 [file Image_2.tif]

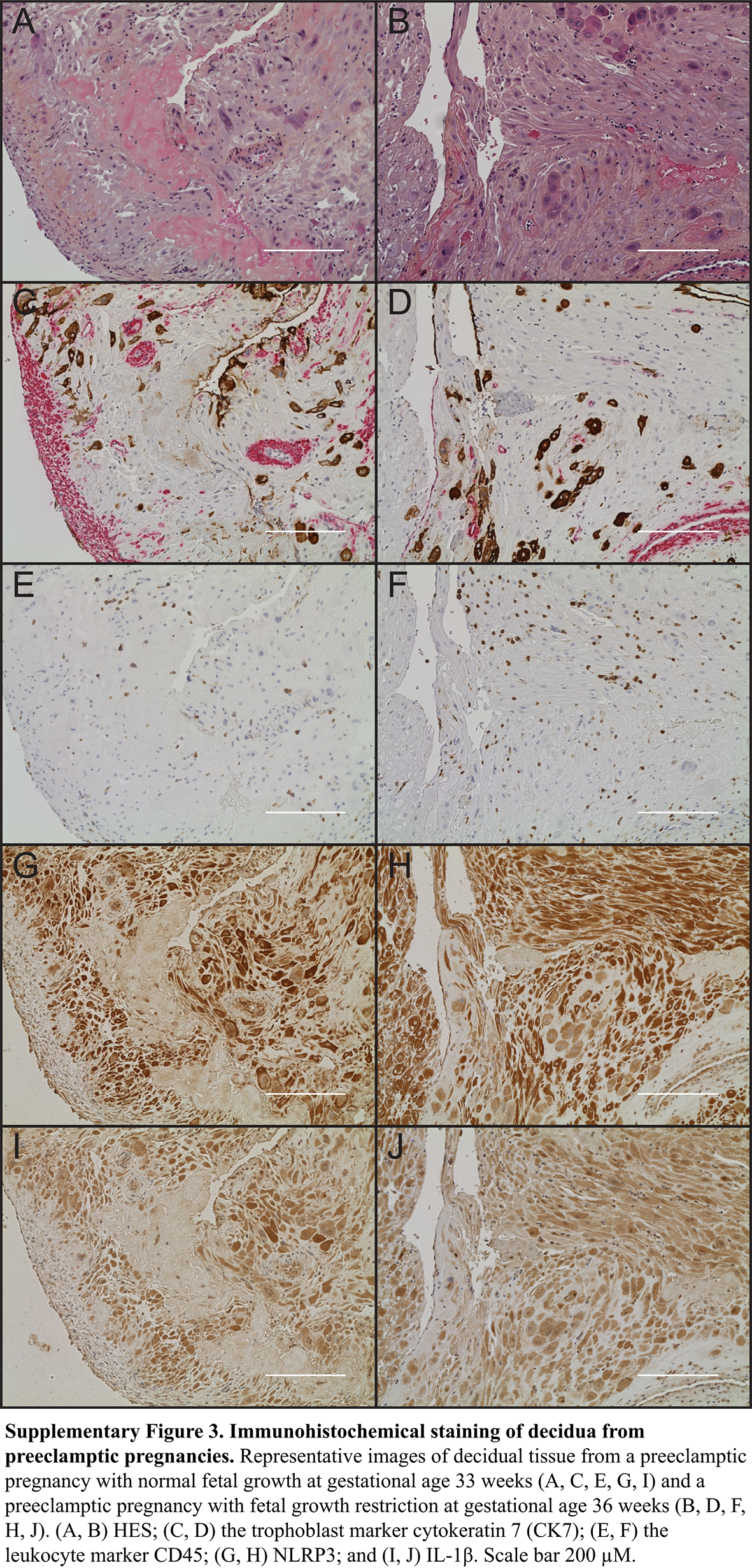

Supplement: Supplementary file 3 [file Image_3.tif]
