# Supplementary material for: Cholesterol Crystals and NLRP3 Mediated Inflammation in the Uterine Wall Decidua in Normal and Preeclamptic Pregnancies
Source: Front Immunol. 2020 Oct 8;11:564712. doi: 10.3389/fimmu.2020.564712 (PMC7578244; doi:10.3389/fimmu.2020.564712)
Supplement: Supplementary file 4 [file Table_1.docx]

Supplementary Material

| **Supplementary Table 1.** Quantitative expression intensities* in the decidua | | | | | | | |
| --- | --- | --- | --- | --- | --- | --- | --- |
|  |  | **Normal pregnancies**  **(n=43)** | | **Preeclampsia without FGR**  **(n=19)** | | **Preeclampsia with FGR**  **(n=28)** | |
|  |  |  |  |  |  |  |  |
|  |  | **Mean** | **SE** | **Mean** | **SE** | **Mean** | **SE** |
| **Total decidual tissue** | |  |  |  |  |  |  |
| NLRP3 | | 0.499 | 0.017 | 0.569† | 0.027 | 0.539 | 0.021 |
| IL-1β | | 0.384 | 0.013 | 0.432†‡ | 0.020 | 0.373 | 0.017 |
| **Areas containing trophoblast** | |  | | | | | |
| NLRP3 | | 0.504 | 0.017 | 0.579† | 0.027 | 0.546 | 0.021 |
| IL-1β | | 0.389 | 0.013 | 0.444†‡ | 0.020 | 0.383 | 0.016 |
| **Areas not containing trophoblast** | |  | | | | | |
| NLRP3 | | 0.483 | 0.018 | 0.535 | 0.028 | 0.520 | 0.023 |
| IL-1β | | 0.363 | 0.013 | 0.395 | 0.020 | 0.364 | 0.017 |
| **Areas containing leukocyte** | |  |  |  |  |  |  |
| NLRP3 | | 0.507 | 0.016 | 0.576† | 0.025 | 0.554 | 0.021 |
| IL-1β | | 0.388 | 0.013 | 0.433 | 0.019 | 0.384 | 0.016 |
| **Areas not containing leukocyte** | |  |  |  |  |  |  |
| NLRP3 | | 0.498 | 0.018 | 0.565† | 0.028 | 0.534 | 0.022 |
| IL-1β | | 0.380 | 0.013 | 0.428†‡ | 0.020 | 0.369 | 0.017 |
| **Areas containing leukocyte and trophoblast** | |  |  |  |  |  |  |
| NLRP3 | | 0.510 | 0.017 | 0.585† | 0.026 | 0.559 | 0.021 |
| IL-1β | | 0.393 | 0.013 | 0.445†‡ | 0.020 | 0.386 | 0.017 |
| FGR, fetal growth restriction; IL-1β, interleukin-1β; NLRP3, Nod-like receptor protein 3; SE, standard error of the mean  * Expression intensity levels are calculated as average staining intensity of all patches given in arbitrary unit  † *P*<0.05 vs normal pregnancies  ‡ *P<*0.05 vs preeclampsia with FGR | | | | | | | |
