# Supplementary material for: Cholesterol Crystals and NLRP3 Mediated Inflammation in the Uterine Wall Decidua in Normal and Preeclamptic Pregnancies
Source: Front Immunol. 2020 Oct 8;11:564712. doi: 10.3389/fimmu.2020.564712 (PMC7578244; doi:10.3389/fimmu.2020.564712)
Supplement: Supplementary file 5 [file Table_2.docx]

Supplementary Material

| **Supplementary Table 2.** Quantitative expression densities* in the decidua | | | | | | | |
| --- | --- | --- | --- | --- | --- | --- | --- |
|  | | **Normal pregnancies**  **(n=43)** | | **Preeclampsia without FGR**  **(n=19)** | | **Preeclampsia with FGR**  **(n=28)** | |
|  |  | **Mean** | **SE** | **Mean** | **SE** | **Mean** | **SE** |
| **Total decidual tissue** | |  |  |  |  |  |  |
| NLRP3 density | | 38.68 | 1.91 | 48.54† | 2.96 | 44.46 | 2.38 |
| IL-1β density | | 32.55 | 1.60 | 40.24† | 2.43 | 34.30 | 2.06 |
| Trophoblast density | | 9.01 | 1.05 | 11.64 | 1.59 | 10.03 | 1.34 |
| Leukocyte density | | 1.95 | 0.17 | 1.56‡ | 0.27 | 2.30 | 0.22 |
| **Areas containing trophoblast** | |  |  |  |  |  |  |
| NLRP3 density | | 39.09 | 1.99 | 49.59† | 3.17 | 43.36 | 2.40 |
| IL-1β density | | 33.80 | 1.73 | 42.94†‡ | 2.68 | 34.78 | 2.19 |
| Trophoblast density | | 16.79 | 1.05 | 18.77 | 1.63 | 14.82 | 1.33 |
| Leukocyte density | | 1.76 | 0.20 | 1.45 | 0.31 | 2.40†§ | 0.25 |
| **Areas not containing trophoblast** | |  |  |  |  |  |  |
| NLRP3 density | | 35.91 | 2.18 | 42.55 | 3.43 | 40.30 | 2.69 |
| IL-1β density | | 28.78 | 1.82 | 33.09 | 2.79 | 31.59 | 2.33 |
| Leukocyte density | | 2.27 | 0.23 | 1.91 | 0.36 | 2.74 | 0.30 |
| FGR, fetal growth restriction; IL-1β, interleukin-1β; NLRP3, Nod-like receptor protein 3; SE, standard error of the mean  * Expression density levels are calculated as the total number of positive pixels divided by the total amount of tissue pixels analyzed (pixels per patch*number of patches)  † *P*<0.05 vs normal pregnancies  ‡ *P<*0.05 vs preeclampsia with FGR  § *P*<0.05 vs preeclampsia without FGR | | | | | | | |
